# Rethinking the sustainability assessment of grass-fed beef: a holistic systematic review

**DOI:** 10.1007/s11250-026-05471-2

**Published:** 2026-11-04

**Authors:** Panashe Paul Mazungunye, Munyaradzi Saruchera, Shepherd Mudavanhu, Kennedy Dzama, Cletos Mapiye

**Affiliations:** 1 Department of Animal Sciences, Stellenbosch University, Stellenbosch, 7600 South Africa; 2 Africa Centre for Inclusive Health Management, Stellenbosch University, Stellenbosch, 7600 South Africa; 3 Department of Agricultural Economics, Stellenbosch University, Stellenbosch, 7600 South Africa

**Keywords:** Animal welfare, Grass-fed beef, Meat quality, Sustainability assessment, Value chain

## Abstract

Consumer demand for grass-fed beef (GFB) is increasing due to its perceived advantages in food safety, healthfulness, animal welfare, and environmental stewardship, yet its sustainability remains contested. This PRISMA-guided systematic review synthesised evidence from 141 studies identified through Web of Science, Scopus, AGRICOLA, and supplementary grey-literature sources to evaluate GFB production, meat quality, and sustainability, and to provide actionable insights for future research, market governance, and policy. The currently inconsistent evidence is insufficient to confirm the sustainability of GFB. Although GFB can offer desirable quality attributes, including leaner carcasses, greater proportions of omega-3 and conjugated linoleic fatty acids, and elevated antioxidant levels, its darker colour and variable tenderness may affect consumer acceptance. Environmentally, reduced grain inputs and greater pasture biodiversity may promote ecological stewardship, but methane emissions and higher land requirements may offset these gains unless adaptive grazing and carbon sequestration are appropriately incorporated. Socially, animal welfare can be enhanced through regenerative grazing practices but can also be compromised when animals are exposed to adverse weather and parasites. Economic performance depends on productivity, certification costs, price premiums, and market access. Despite their growing social relevance, animal welfare, traceability, authenticity, and certification remain insufficiently integrated into sustainability assessments. Overall, the current evidence is fragmented and dominated by farmgate economic and greenhouse gas metrics. Multidimensional, value-chain-based, and context-sensitive sustainability assessment frameworks are proposed to guide credible standards, targeted incentives, and market development.

**Supplementary Information:**

The online version contains supplementary material available at https://doi.org/10.1007/s11250-026-05471-2.

## Introduction

Global demand for grass-fed beef (GFB) is increasing. GFB is arguably defined as meat from free-roaming cattle that primarily graze on forages throughout their lives (Bauman and Williams 2021). This trend is largely driven by growing concerns about animal welfare, human health, and the environmental impacts of grain-fed beef production (Kumar et al. 2022). The global GFB market was valued at approximately US$ 13.56 billion in 2025 and is projected to reach US$ 21.23 billion by 2035 (Future Market Insights 2026). However, these estimates should be interpreted cautiously because they depend on varying market definitions, forecasting assumptions, climatic conditions, and regional contexts. Such regional variation is reflected in the contribution of GFB to national beef supplies, ranging from about 4% in the United States of America (USA; Bauman and Williams 2021), compared with 60–70% in Australia (Thomas et al. 2021), and more than 80% in Brazil (Agethen et al. 2024).

In many low-income tropical countries, GFB production remains dominant because limited grain availability and feedlot infrastructure constrain the adoption of intensive finishing systems (Rubio Lozano et al. 2021). GFB also aligns with consumer preferences for meat perceived as healthier and more humanely produced. Grazing allows cattle to express natural behaviours, while GFB is typically leaner and richer in health-promoting omega-3 (n-3) polyunsaturated fatty acids (PUFA), conjugated linoleic acids, and antioxidants (Broom 2021; Ponnampalam et al. 2024a; Peripolli et al. 2025). However, these nutritional and welfare advantages involve environmental trade-offs. Well-managed grazing can enhance biodiversity and soil carbon sequestration (Rowntree et al. 2020), but the magnitude and persistence of these benefits are dependent on climatic conditions, pasture quality, stocking intensity, and grazing management (Rowntree et al. 2016; Broom 2021). Conversely, slower animal growth and lower land-use efficiency may increase production costs, land requirements, and methane (CH_4_) emissions per kilogram of beef, potentially offsetting the gains from biodiversity enhancement and carbon sequestration (Molidor 2017; Tichenor et al. 2017b; Wróbel et al. 2023). Hence, GFB sustainability is context-dependent and requires an integrated evaluation of nutritional, welfare, economic, and environmental outcomes.

Evidence on the sustainability of GFB remains uncertain (Hayek and Garrett 2018; Klopatek et al. 2022a), largely because the concept lacks a universally accepted definition. In practice, GFB systems vary in forage composition, supplementation strategies, animal health interventions, and the use of growth promotants. Moreover, not all GFB is certified, and some regulatory frameworks classify beef as grass-fed when only a minimum proportion (e.g., ≥ 50%) of lifetime feed intake is forage-based rather than exclusively forage-fed (Cheung and McMahon 2017). This heterogeneity groups together production systems with different feeding regimes, management practices, product attributes, and certification requirements, limiting the comparability and interpretation of sustainability assessments. Existing assessment frameworks further compound this challenge by lacking methodological consistency, multidimensional coverage, and integration of value-chain interactions, trade-offs, feedback loops, and system dynamics. Consequently, holistic insight, Consequently, holistic insight, evidence-based prioritisation and policy development for sustainable value creation across GFB systems remain constrained. This systematic review therefore adopts a value-chain perspective, while acknowledging the predominance of farm-level evidence, to synthesise the sustainability outcomes of GFB production and identify actionable pathways for future sectoral development.

## Definitions, standards, and regulations of grass-fed beef

GFB production systems rely on forage-based diets (i.e., grasses, legumes, and shrubs) throughout the production cycle, with cattle primarily grazing pastures, typically supplemented with conserved forages such as hay or silage. However, GFB lacks a universally accepted definition, leading to wide variation in production practices, reported carcass attributes, and sustainability outcomes (Stampa et al. 2020; Krusinski et al. 2022b). Terms such as ‘extensive’, ‘free-range’, ‘outdoor-reared’, ‘grass-finished’, and ‘pasture-raised’ are often used interchangeably with grass-fed, although they differ in emphasis. Certified ‘grass-finished’ refers to cattle finished exclusively on forage, even if grains were consumed earlier (Krusinski et al. 2022b). ‘Extensive’ systems generally use large land areas with lower inputs and stocking rates, although exceptions exist in some Southern African smallholder systems, where stocking rates are high (Marandure et al. 2018). ‘Free-range’ and ‘outdoor-reared’ focus on outdoor access rather than diet, whereas ‘pasture-raised’ implies that the beef herd obtains most or all nutrition from forages on pasture, with confinement limited to situations such as calving, routine management, and protection from adverse weather and predators (Conner and Oppenheim 2008). Some systems also use ‘grass feedlots’, where cattle are confined but fed harvested forages (Cheung and McMahon 2017).

Third-party certification has become a key mechanism for verifying the authenticity and traceability of GFB. Reflecting consumer preference for beef marketed as grass-fed, hormone-free, and antibiotic-free, certification schemes typically prohibit routine grain supplementation and restrict synthetic growth-promoting substances, including anabolic hormones, beta-agonists, ionophores, and sub-therapeutic antibiotics (Poore et al. 2020). Grain supplementation during emergencies and therapeutic antibiotic use during illness may be permitted to safeguard animal welfare. However, differences in definitions, exemptions, and enforcement generate considerable inconsistencies among schemes. As private standards often exceed national requirements for feeding, medication, growth promotants, and confinement, compliance does not necessarily indicate comparable production practices. Despite the growing private-sector scrutiny of GFB governance, regulatory fragmentation continues to hinder consistent comparisons across schemes and countries. Thus, certification should not be interpreted as a uniform guarantee of GFB production practices but as a scheme-specific claim whose credibility and comparability depend on transparent definitions, harmonised standards, and independent verification.

The standards presented in Table 1 demonstrate that GFB encompasses substantially different combinations of dietary, animal health, and confinement requirements. Australia’s Pasturefed Cattle Assurance System (PCAS) requires continuous pasture access and prohibits grain feeding; however, antibiotic and hormone use are optional (Coombs et al. 2021). Similarly, the former United States Department of Agriculture (USDA) grass-fed standard focused on forage diets and continuous pasture access but was abandoned in 2016 due to poor adoption and enforcement, as well as partial claims (e.g., 50% grass-fed), leaving the USA without a national GFB standard (Cheung and McMahon 2017). In response, organisations such as the American Grassfed Association introduced stricter criteria, banning the use of grains, antibiotics, and hormones (American Grassfed Association 2017). In the European Union, Irish standards require at least 90% of lifetime forage intake, and a minimum of 220 grazing days per year, allowing limited grain feeding (Bord Bia 2020). In contrast, the Pasture for Life (2025) standards only allow forage diets and the non-confinement of animals. The Grassfed Association of South Africa prohibits the use of grains and growth promotants, whereas the Greenfields protocol permits grain supplementation of up to 5% of lifetime dry matter intake under harsh production conditions (Bay Meat Market, 2025). However, broader industry practice may still classify cattle that consume limited amounts of grain (≤ 500 kg) over their lifetimes as grass-fed (Müller 2018; SAMIC 2019). The inclusion of studies allowing grain intake (e.g., 500 kg lifetime) could compromise the pure grass-fed benchmark by introducing nutritional confounding factors that alter rumen metabolism, animal growth, carcass characteristics, and fat deposition. Such inclusion weakens the external validity of grass-fed systems and biases quality metrics toward grain-fed physiology. Thus, such studies warrant stratified analyses or exclusion to preserve scientific interpretability.

**Table 1:** Differences in standards of grass-fed beef across certification bodies

Practice	PCAS^a^ (Australia)	USDA^b^ (USA)	AGA^c^ (USA)	Pasture for Life (UK^d^)	Bord Bia (Ireland)	GFASA^e^ (South Africa)	Greenfields (South Africa)
Antibiotic use	Optional	Optional	No	Only for disease treatment	No	No routine use	No feed grade
Synthetic hormone use	Optional	Optional	No	No	No	No	No
Ionophores	Optional	Optional	No	No	No	No	No
Confinement	No	No	No	No	Yes	No	No
Grain supplements	No	No	No	No	Yes	No	Yes

^a^ Pasturefed Cattle Assurance System, ^b^ United States Department of Agriculture, ^c^ American Grassfed Association,

^d^ United Kingdom, ^e^ Grassfed Association of South Africa

In this review, GFB refers to meat from cattle raised on a forage-based (100%) diet from weaning, with continuous access to natural (rangeland), semi-natural, or cultivated pastures, and without the use of growth promotants. GFB production systems use diverse fresh forages, including grasses, legumes, shrubs, and forbs, as well as conserved forages such as hay and silage, whose botanical composition, nutrient and bioactive-compound profiles, digestibility, intake potential, and seasonal availability vary substantially among systems and may consequently influence meat quality and sustainability outcomes. Cattle raised under these conditions are not permanently confined and may receive limited grain supplementation only in exceptional circumstances, such as drought, illness, or other emergencies. Therapeutic antibiotics are allowed when needed to support animal health and welfare. The operational definition was developed to integrate core certification requirements while ensuring the representativeness of existing standards, contextual adaptability, and practical guidance for evidence-informed policy and implementation across diverse global contexts.

## Methodology

### Data search and eligibility criteria

The review follows the Preferred Reporting Items for Systematic Reviews and Meta-Analyses (PRISMA) guidelines (Page et al. 2021) to identify, screen, and select relevant literature on GFB. Literature searches were conducted in the Web of Science, Scopus, and Agricola electronic databases to obtain peer-reviewed scholarly articles. The search was limited to English studies from the year 2000 onwards. Electronic database searches were supplemented by citation searches, industry reports, and other searches using the Google search engine. Grey literature broadens systematic reviews by capturing relevant studies missed in database searches and enabling a more comprehensive and balanced synthesis (Desiderio et al. 2022). The initial literature search was conducted on 1 June 2025, followed by updates on 20 December 2025, 1 May, and 10 September 2026 using the same search strings.

To obtain a holistic perspective on the sustainability of GFB systems, the search strategy was designed to capture a broad range of studies on GFB. A broad Boolean search strategy was first developed to capture literature on GFB: (“grass-fed” OR “grass-finished” OR “pasture-fed” OR “pasture-finished” OR “forage-fed” OR “forage-finished”) AND (beef OR steer OR suckler OR bull OR cattle OR heifer OR cow). Subsequently, this core search string was combined with additional terms related to specific aspects of GFB, such as production, consumption, marketing, quality, contributions, challenges, and opportunities (Table 2). The database-specific search strings are provided in the supplementary material (Table S1).

**Table 2:** Search terms used to identify literature on grass-fed beef

Aspect	Search terms
Production	production OR supply AND “management practices” AND perceptions
Consumption	consumption OR demand OR “willingness to pay” AND perceptions
Marketing	market* OR “marketing practices”
Quality	quality OR “credence attributes”
Contribution/Sustainability attributes	“environmental contribution” OR “environmental impact” OR “environmental consequences” OR “environmental assess*” OR “social impact” OR “social contribution” OR “economic contribution” OR “economic impact” OR “economic performance” OR sustainability
Challenges/opportunities	challenges OR opportunities

### Data eligibility criteria

**Fig. 1:**
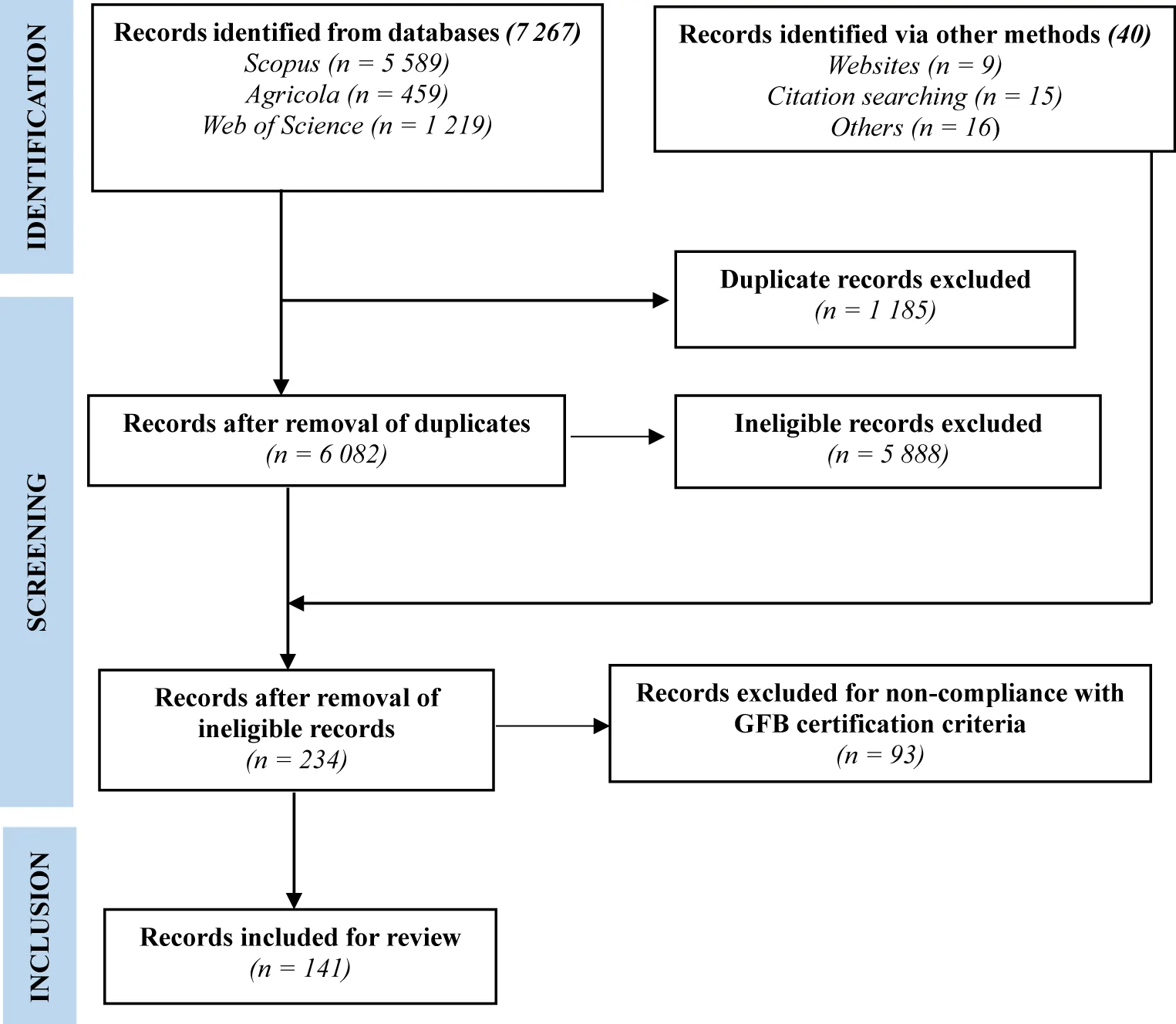
PRISMA flow diagram showing records (n) identified, screened, and included in the review

The database search yielded 7 267 records. The inclusion and exclusion criteria presented in Table 3 were applied during screening for relevance, following the PRISMA procedure shown in Fig. 1. For eligible studies, when full texts were not accessible via database links, interlibrary loans and author requests via ResearchGate were used. Snowballing was also applied through reference-list screening and forward citation tracking.

### Data analysis

Evidence was synthesised narratively in two stages. First, the production practices and marketing dynamics of GFB were summarised. Second, beef quality evidence was classified into intrinsic, extrinsic, and credence attributes and interpreted against economic, environmental, and social sustainability dimensions. The synthesis also reviewed empirical sustainability assessments of GFB production systems, and identified key limitations. Although direct comparisons with grain-fed beef were not a primary objective, relevant comparative findings were included to contextualise GFB within dominant production systems. The results are presented in the following sections, covering production and marketing aspects, followed by an examination of GFB quality attributes.

**Table 3:** Eligibility criteria for the systematic review

Inclusion criteria	Exclusion criteria
Publications focused on beef cattle production systems described as grass-fed, grass-finished, pasture-fed, pasture-finished, forage-fed, forage-finished, or pasture-raised, and met the operational definition of GFB used in this review.	Studies focused exclusively on grain-fed, feedlot-based, concentrate-fed, dairy, non-bovine, or undefined production systems without comparison to GFB production systems, or studied systems did not meet the operational definition of GFB used in this review.
Studies examining at least one aspect of GFB, including production, consumption, marketing, meat quality, sustainability, challenges, and opportunities.	Studies addressing topics relevant to this review, e.g., meat quality, production, sustainability, etc., without an explicit link to grass-fed or pasture-based beef systems.
Peer-reviewed studies reporting original empirical findings relevant to the review topic, and providing sufficient methodological and results-based information for evaluation and data extraction.	Duplicate records, editorials, commentaries, conference abstracts, and studies with insufficient methodological or results-based information.
Studies available in full text, published in English, and published from 2000 onwards.	Studies unavailable in full text, not published in English, or published before 2000.

### Risk of bias evaluation

A formal risk-of-bias assessment was not undertaken because the evidence spanned experimental studies, life cycle assessments, economic analyses, surveys, reviews, industry reports, and policy documents, precluding the use of a single valid tool. Substantial variations in aims, designs, contexts, methods, and sustainability indicators further limited comparability, certainty, transferability, and generalizability. However, the procedures described by Desiderio et al. (2022) were applied to reduce bias. These include: (1) unrestricted searches by design, method, or geography; (2) searches of multiple databases and grey literature; (3) consideration of all eligible evidence; (4) explicit acknowledgement of heterogeneity; and (5) transparent synthesis of convergent and divergent findings.

## Overview of production and marketing of grass-fed beef

### Production practices

Reliable estimates of GFB contributions to national beef supplies remain scarce because definitions vary, studies are costly, and official data seldom disaggregate production systems, especially in low- and middle-income countries. The available evidence suggests that forage-based grazing systems, in which ≥ 90% of dry matter intake is forage, produced about 34% of global beef in 2015 (Gerber et al. 2015), suggesting that the share from strictly 100% GFB systems is lower. Nevertheless, GFB predominates in sub-Saharan Africa and major beef-producing countries, including Brazil, Australia, Argentina, New Zealand, and Uruguay (Greenwood 2021). Reported GFB shares are > 80% in Brazil, 60–70% in Australia, 72% in Argentina, and ~ 85% in Uruguay (Thomas et al. 2021; Agethen et al. 2024; El Instituto Nacional de Carnes 2024). Although estimates are missing for Africa, smallholders in arid regions largely rely on forages, while those in humid areas supplement with crop residues (Mapiye et al. 2020).

GFB production systems are diverse, ranging from natural to cultivated pastures composed of grasses, legumes, shrubs, and forbs (Poore et al. 2020; Klopatek et al. 2022a). Silvopastoral systems integrating shrubs and trees in pastures are common in Brazil and Australia (Broom 2019). Rotational grazing, commonly used in certified GFB production systems, can enhance soil health, forage biomass, and composition, thereby increasing meat yield and quality (Poore et al. 2020). Finishing beef cattle on forage takes 18–48 months, depending on agroecology, breed, target market, and forage quantity and quality (Chingala et al. 2017; Rubio Lozano et al. 2021). Because grain supplementation, growth promotants, and antibiotics are restricted, breed choice is central. Indigenous, locally synthesised breeds, and their hybrid genotypes, often show adaptive grazing behaviours and resilience suited to both temperate and tropical environments (McIntosh et al. 2023). For example, *Bos indicus* and *Bos taurus africanus* breeds indigenous to the tropics tolerate heat, drought, and parasites, enabling low-input forage-based production aligned with GFB standards (Mapiye et al. 2020; McIntosh et al. 2023). However, they grow more slowly and produce lighter carcasses than late-maturing temperate breeds (Mapiye et al. 2020).

### Marketing of grass-fed beef

Global GFB demand is rising due to perceived health, safety, animal welfare, and sustainability advantages, and lower reliance on hormones and antibiotics (Nogoy et al. 2022; Davis et al. 2022). In 2020, the USA was estimated to contribute approximately half of the global growth in the GFB market by 2024 (Technavio 2023), supported by an annual demand growth of nearly 20% and the willingness of 20–30% of consumers to pay premium prices (Qushim et al. 2018). However, a substantial share of this demand is currently met through Australian imports. Consumer preferences for GFB vary by country. USA consumers prioritise products from natural pastures with no hormones or antibiotics, while Europeans favour local GFB, and the Chinese prefer domestic products (Lim et al. 2018; Poore et al. 2020; Fu et al. 2024). These trends are largely driven by availability, affordability, and adaptability. Marketing channels include direct sales, online sales, abattoirs, supermarkets, restaurants, and farmers’ markets (Lim et al. 2018). Direct marketing is preferred in the USA for its ability to provide better compensation and foster customer loyalty (Gwin et al. 2012; Poore et al. 2020). Consumer knowledge of the provenance, nutritional attributes, and health benefits of GFB increases their willingness to pay, underscoring the importance of communicating product information through clear labelling and targeted marketing strategies. Therefore, a detailed understanding of the quality attributes driving these preferences is essential (Santos et al. 2021).

## Quality attributes of grass-fed beef

Beef quality is complex and multidimensional, comprising both intrinsic and extrinsic attributes (Henchion et al. 2014). Intrinsic attributes are the inherent structural and biochemical properties of meat, including nutritional composition, colour, fat content, tenderness, and flavour, which cannot be altered without changing its biological properties. Extrinsic attributes, such as price, packaging, provenance, expiry date, certification, and sustainability claims, function as external information cues that may change without modifying the meat. These attributes are further grouped into search, experience, or credence cues (Henchion et al. 2014). Search cues, such as colour, visible fat, and price, are evaluated through inspection at the time of purchase. Experience cues, particularly tenderness, juiciness, and flavour, are assessed during consumption and strongly influence satisfaction and repeat purchases. Credence cues, including perceived healthfulness, nutritional value, animal welfare, provenance, and sustainability, require trustworthy supporting information, often through certification or labelling. Because stakeholders prioritise these various cues differently, their integration is essential for evaluating GFB quality and aligning value chain expectations. Considering this background, intrinsic quality evidence will be discussed, followed by extrinsic quality and sustainability-related attributes and assessment methods.

### Colour of grass-fed beef

#### Lean colour

Beef colour, a key driver of consumer purchasing decisions (Perry et al. 2019), primarily reflects the concentration and oxidation state of myoglobin, which are mostly influenced by genetics, diet, physical activity, muscle type, postmortem handling, chilling, packaging, and storage (Poveda-Arteaga et al. 2023). When myoglobin is in its oxygenated ferrous form (oxymyoglobin), it produces a bright cherry-red colour, commonly observed in grain-fed beef and associated with freshness (Perry et al. 2019). In contrast, GFB often appears darker, partly because pasture-finished cattle are typically slaughtered at an older age and experience greater physical activity, both of which increase muscle myoglobin content and the proportion of oxidative muscle fibres (Apaoblaza et al. 2020; Poveda-Arteaga et al. 2023). In addition, lower subcutaneous and intramuscular fat in GFB also reduces the lightness (L^*^) values of lean colour, contributing to a darker visual appearance (Priolo et al. 2001; Duarte et al. 2022). Consumers often perceive this darker colour as an indicator of spoilage, which can lower acceptance (Perry et al. 2019). However, GFB is rich in antioxidants (i.e., α-tocopherol and β-carotene), which may prevent myoglobin oxidation and maintain redness for extended periods (Legako et al. 2018; Duarte et al. 2022).

#### Fat colour

GFB fat tends to be yellower than the typically whiter fat of grain-fed beef because fresh grass contains carotenoids (i.e., yellow-orange pigments such as β-carotene and lutein) that accumulate in adipose tissue through pathways not yet fully understood (Daley et al. 2010). Dietary carotenoid and energy intake, feeding duration, and animal genetics influence carotenoid deposition and concentrations in adipose tissue (Dunne et al. 2009). Older pasture-reared suckler cows often have more yellow fat because the prolonged intake of carotenoid-rich forage promotes the progressive deposition of carotenoids in adipose tissue over time (Dunne et al. 2009). The lactation-induced negative energy balance may further intensify fat yellowness through the repeated mobilisation of triglycerides from adipocytes, thereby concentrating residual fat-soluble carotenoids in the remaining lipid depots (Dunne et al. 2009). Similarly, during periods of feed restriction, the mobilisation of adipose reserves increases carotenoid concentration and fat yellowness, although this effect may be reversed if animals subsequently restore their fat reserves (Dunne et al. 2009). Consumer preferences for yellow fat vary by region. For example, Korean consumers showed no preference between yellow and white fat, whereas many markets preferred the whiter fat typical of grain-fed beef (Doyle et al. 2023; Kim et al. 2023). In South Africa, 43.74% of consumers preferred white fat (due to lack of knowledge about yellow fat), 13.59% preferred yellow fat, and 42.68% were indifferent (Maré et al. 2013). Despite mixed preferences, yellow fat can serve as a GFB biomarker and a marketing tool for its potential health benefits.

#### Bioactive profile

The nutritional and health profiles of beef are primarily determined by the animal’s diet, which strongly influences the level of intramuscular fat, fatty acid (FA) composition, and other bioactive compounds (Krusinski et al. 2022b; Ponnampalam et al. 2024a). The succeeding subsections discuss the FA, vitamin, and polyphenolic compositions of GFB.

#### Fatty acid content and composition

GFB is often marketed as a leaner and healthier option for health-conscious consumers seeking to reduce their intake of saturated FA and cholesterol, which are associated with cardiovascular disease risk (Poore et al. 2020; Xu et al. 2023). Studies consistently report that GFB has a lower total fat content than grain-fed beef (Fruet et al. 2018; Butler et al. 2021; Krusinski et al. 2022a), with Duckett et al. (2013) reporting up to 61% less. In some contexts, finishing duration and animal slaughter age influence outcomes, as short-term finished cattle (Ponnampalam et al. 2006; Klopatek et al. 2022b) or younger grain-fed cattle (Hall et al. 2016) may also exhibit low fat content. Lean beef commands premium prices in the EU and South Africa (Fisher 2007; Chingala et al. 2017), whereas carcass valuation systems in the USA, Australia, and Japan favour higher intramuscular fat levels (Orime et al. 2012; Mathews and Johnson 2013; Coombs et al. 2021). Consequently, some USA producers finish GFB to higher intramuscular fat contents (Poore et al. 2020).

GFB typically contains higher proportions of polyunsaturated FA (PUFA), and up to 62% lower total saturated FA such as myristic (14:0) and palmitic (16:0) acids (Nogoy et al. 2022; Peripolli et al. 2025), which have been associated with negative health effects (Xu et al. 2023). It is also richer in n-3 PUFAs and biohydrogenation intermediates (e.g., rumenic, trans-vaccenic, and palmitoleic acids), which are associated with anti-inflammatory and cardiovascular benefits (Butler et al. 2021; Xu et al. 2023; Ponnampalam et al. 2024a). Fresh forages are associated with the higher PUFA proportions in GFB, as they are high in α-linolenic acid (ALA), a precursor of long-chain n-3 PUFAs. However, elevated PUFA levels in meat increase its susceptibility to oxidation, although the relatively high antioxidant content of GFB may mitigate these effects (Ponnampalam et al. 2024b), as explained in the next sections.

#### Vitamins

Regardless of diet, beef is a good source of vitamins A, B6, B12, D, and E (Daley et al. 2010). However, cattle on fresh forages produce beef with up to nine times more β-carotene (provitamin A) than grain-fed beef (Duckett et al. 2013). GFB also contains higher levels of vitamin E (α-tocopherol), approximately three times those of grain-fed beef (Duckett et al. 2013). These vitamins act as potent antioxidants, enhancing oxidative stability and delaying the oxidation of myoglobin, lipids, and proteins during retail display (Legako et al. 2018; Duarte et al. 2022). This resistance to oxidation makes GFB more suitable for further processing (Farouk and Wieliczko 2003). In addition, vitamins A, B6, B12, D, and E contribute to human health by supporting vision, bone health, metabolic processes, and the functioning of the immune and nervous systems (Daley et al. 2010; van Vliet et al. 2021).

#### Phytochemicals

Cattle grazing on diverse plant species produce beef with higher concentrations of phytochemicals, including polyphenols, carotenoids, and essential oils, than grain-fed beef (van Vliet et al. 2021; Peripolli et al. 2025). These dietary phytochemicals display antimicrobial, antioxidant, and immunomodulatory properties in both animals and meat consumers (Provenza et al. 2019; Kazemi and Ariapour 2025). Their antioxidant properties reduce the susceptibility of beef to oxidation and colour deterioration (Ponnampalam et al. 2024b). Beyond the shelf-life benefits of phytochemical transfer from grazed plants to meat, flavonoid-, saponin-, and essential oil-rich rangeland forages may enhance ruminant digestibility and performance and reduce methane emissions, thereby improving the meat yield and environmental sustainability of GFB (Kazemi and Ariapour 2025).

### Nutritional relevance and potential human health implications

Consumers perceive GFB as healthier due to its naturally enhanced bioactive profile. Many populations do not achieve the recommended long-chain n-3 intakes, particularly where oily fish consumption is low, and GFB can contribute to dietary n-3 intake in such contexts (McAfee et al. 2011; Butler et al. 2021). Existing evidence indicates that substituting GFB for grain-fed beef can increase intake and *de novo* synthesis of n-3 PUFA in consumers who regularly eat red meat (McAfee et al. 2011). Consumers may also perceive GFB as healthier because its n6:n‑3 ratio often falls below the recommended 4:1 threshold associated with anti-inflammatory dietary benefits (Daley et al. 2010). However, this ratio as a health marker has been criticised (Provenza et al. 2019), thus warranting further investigation. Notably, beef is a rich source of biohydrogenation intermediates, including rumenic, trans-vaccenic, and palmitoleic acids, which seem to confer human health benefits (Xu et al. 2023; Ponnampalam et al. 2024a). Clinical trials remain limited, and further human studies are necessary to establish if the compositional advantages of GFB yield tangible health benefits (Davis et al. 2022).

### Eating quality

#### Tenderness

Tenderness is a key determinant of consumer satisfaction, willingness to pay premiums and repeat purchases (Roy and Bruce 2023). Although earlier reviews reported that GFB is generally tougher than grain-fed beef (Daley et al. 2010; Santos et al. 2021), the evidence in this review is inconsistent. Several studies found no significant differences in tenderness (Carrillo et al. 2016; Fruet et al. 2018; Duarte et al. 2022), while others reported GFB as tougher (Antonelo et al. 2022; Gomez et al. 2022). In contrast, studies from Uruguay and Chile reported GFB as more tender than grain-fed beef (del Campo et al. 2008; Morales et al. 2015).

Tenderness is primarily influenced by collagen crosslinking and solubility, sarcomere length and postmortem proteolysis, intramuscular fat, and cooking-induced protein denaturation (Roy and Bruce 2023). Variation in these mechanisms is strongly associated with, and may be confounded by, growth rate, slaughter age, carcass weight, and fatness. Because grass-fed cattle generally grow more slowly and are slaughtered later, their beef is often leaner and less tender. As the animal gets older, the content of mature collagen cross-links increases, thereby reducing tenderness (Roy and Bruce 2023). Reduced intramuscular fat in GFB may also contribute to lower tenderness, whereas adipocyte expansion can disrupt collagen structure and improve tenderness (Carrillo et al. 2016; Roy and Bruce 2023). However, when GFB and grain-fed cattle are finished at a similar age or fat endpoint, tenderness differences may disappear (Faucitano et al. 2008; Duckett et al. 2013). In one study, subtropical Hawaiian pasture-finished cattle showed growth rates and tenderness comparable to those of grain-fed cattle, despite a shorter growing period and lighter carcasses (Kim et al. 2012). Higher α-tocopherol levels in GFB may also enhance collagen turnover via matrix metalloproteinases, contributing to improved GFB tenderness in some settings (Purslow et al. 2012).

#### Flavour

GFB generally has a flavour that is distinct from that of grain-fed beef (Daley et al. 2010). The distinct flavour of GFB is attributed to its elevated PUFA content, particularly ALA, as well as higher concentrations of triterpenoids, phenols, and other secondary metabolites (Arshad et al. 2018). Panellists have described GFB as ‘earthy’, ‘grassy’, ‘gamey’, ‘fishy’, ‘rancid’, ‘sour’, ‘bitter’, or having some off-flavour, with ALA being linked to the grassy or fishy notes (Arshad et al. 2018; Moholisa et al. 2019; Duarte et al. 2022). However, findings remain inconsistent. Brazilian panellists rated grain-fed beef higher for off-flavour, while Irish studies reported no flavour differences; unlike trained panellists, consumers often do not detect off-flavours (Fruet et al. 2018; Moloney et al. 2022).

#### Juiciness

In contrast to Santos et al. (2021), this review found no consistent juiciness differences between GFB and grain-fed beef when animals were finished to similar fat endpoints or carcass weights (Faucitano et al. 2008; Moloney et al. 2022), or even with older grass-fed cattle (Moholisa et al. 2019). Any perceived increase in juiciness may reflect greater saliva secretion during mastication of tougher, collagen-rich meat rather than a true difference in water retention (Moholisa et al. 2019).

#### Overall acceptance

Despite possible differences in tenderness and flavour, GFB is preferred by some consumers. Acceptance is strongly shaped by culture, familiarity with products, and market context. In tropical regions, GFB is widely accepted on cultural and familiarity grounds (Rubio Lozano et al. 2021). In Brazil, 42% of consumers prefer GFB, compared with 24% for grain-fed beef (Mottin et al. 2019). Similarly, native and rural consumers in South Africa and Botswana tend to prefer chewy, strongly-flavoured beef suited to slow-cooking methods, whereas urban consumers prefer more tender meat (Chingala et al. 2017). In grain-fed-dominant markets, GFB is often positioned as a premium niche product for health-, environmental-, and ethics-oriented consumers (Stampa et al. 2020; Peripolli et al. 2025). Acceptability may be improved through quality-differentiated branding and “grass-fed” labelling, which influence consumer perceptions, as well as through post-slaughter ageing, particularly dry-ageing, which improves tenderness and flavour (Berger et al. 2018; Antonelo et al. 2022). In some contexts, short-grain supplementation during finishing may improve eating quality without compromising the beneficial FA profile (Fruet et al. 2018). However, its effects on FA composition may vary depending on dietary and production conditions (Ponnampalam et al. 2006).

### Environmental quality

#### Land use

As land scarcity intensifies under population growth and climate change, claiming GFB as land-wasting and grain-fed beef as land-saving is overly simplistic and misleading (Mathews and Johnson 2013). GFB can support producer livelihoods while utilising marginal, less fertile, or erosion-prone land unsuitable for cropping, converting human-inedible forage into nutrient-dense food (McCutcheon et al. 2015; Oerly et al. 2022; Castaño-Sánchez et al. 2023). However, compared with grain-fed systems, GFB often requires more land and longer production periods to achieve equivalent beef output because grain-based diets are more energy-dense and support faster growth and heavier carcasses (Hayek and Garrett 2018; McGee et al. 2023). This creates a trade-off between utilising larger areas of lower-productivity land and optimising production efficiency.

Evidence suggests that land-use outcomes are highly context-dependent. Hayek and Garrett (2018) estimated that existing USA pastures and forage cropland could support only 61% of current beef consumption under complete grass finishing. However, Broom (2019) reported that grazing systems based on fertilised or irrigated pastures may have land-use requirements comparable to grain-fed systems, whereas extensive unimproved pasture systems require substantially more land. Semi-intensive silvopastoral systems, by contrast, can achieve the lowest land-use demand among the systems compared. Thus, comparisons should account for the entire production system, including pre-feedlot management of weaners, feed production intensity, finishing duration, and the use of agro-industrial co-products, which can improve resource efficiency. Overall, land-use sustainability in GFB reflects context-specific trade-offs between production efficiency and the ecosystem services provided by grazing landscapes.

#### Land degradation

Cattle production can degrade land, but impacts differ between pasture-based and feedlot systems. In some beef value chains, the most extensive land-use change is deforestation for pasture and forage expansion. In the Brazilian Amazon, cattle ranching accounts for about 80% of forest clearing, especially in remote areas where governance is weak and indirect supply-chain links obscure accountability (Skidmore et al. 2021). In GFB systems, degradation risk is greatest where stocking rates exceed ecological carrying capacity. Overstocking, continuous grazing, and/or fertiliser use can simplify plant communities, compact soils, accelerate erosion, reduce biodiversity, and undermine long-term profitability (Cheung and McMahon 2017; Molidor 2017). However, well-managed rotational or holistically planned grazing can support vegetation recovery, improve soil structure and water retention, increase forage productivity, and enhance ecosystem resilience when rest periods are adequate (Cheung and McMahon 2017; Teague and Kreuter 2020).

Feedlot production reduces the grazing land footprint and broad-acre erosion but concentrates impacts locally, including soil compaction, pen-surface degradation, reduced biotic integrity, and pathogen-contaminated dust (McGinn et al. 2003; Greenwood 2021; Broom 2021). Feedlots also shift land-use pressures upstream through their dependence on feed crops. Crop cultivation for concentrates can lead to soil disturbance, erosion, loss of soil organic carbon, fertiliser-related nitrous oxide (N_2_O) emissions, agrochemical pollution, and water-quality decline (Oerly et al. 2022). Overall, evidence suggests that feedlots may reduce on-site land degradation while externalising impacts to croplands. In contrast, GFB production systems can either regenerate or degrade landscapes, depending on grazing intensity, recovery time, ecological context, and governance.

#### Ecosystem services

Well-managed GFB production can provide important ecosystem services, particularly in semi-natural grasslands that have co-evolved with livestock grazing over centuries (Kleppel and Frank 2022). Unlike grain-fed production systems, GFB production systems may help maintain open landscapes and contribute to biodiversity conservation, habitat preservation, water filtration, carbon sequestration, soil protection, and reduced risks of air and water pollution (McCutcheon et al. 2015; Provenza et al. 2019; Teague and Kreuter 2020). Although grain-fed systems also generate some of these benefits during their grazing phase, the advantages diminish as animals spend less time on pasture.

In regions with extensive natural grasslands, such as Brazil, well-managed grazing can play a key role in restoring and conserving degraded ecosystems (Lobato et al. 2014). Adaptive grazing, including regenerative or rotational systems with controlled burning, can maintain species-rich grasslands, control woody and invasive alien plants, and create sward structures that promote seed germination (Schuman et al. 2002; Kleppel and Frank 2022; Wróbel et al. 2023). Furthermore, it can strengthen livestock-ecosystem interactions by enhancing seed dispersal, creating microhabitats, improving nutrient cycling, and promoting herbaceous plant growth (Krusinski et al. 2022b; Wróbel et al. 2023).

Livestock excreta can increase soil nitrogen in rangelands, enhancing biomass production and microbial processes that support soil organic matter, moisture retention, and carbon sequestration (Schuman et al. 2002; Molidor 2017). These benefits may vary with grazing management and the intensity of nitrogen fertiliser application. Specifically, moderate nitrogen fertilisation may support soil microbial and plant growth, whereas excessive inputs can alter soil chemistry, disrupt plant-microbe interactions, increase N_2_O emissions, and elevate risks of nutrient leaching and eutrophication (Kleppel and Frank 2022). The literature indicates that GFB production systems may enhance ecosystem services only when grazing pressure, nutrient inputs, and governance measures are carefully managed to balance environmental benefits with trade-offs in emissions, biodiversity, and productivity.

#### Greenhouse gas emissions

Beef production is a major contributor to climate change, accounting for over 60% of anthropogenic greenhouse gas (GHG) emissions from ruminants (FAO 2023). Emissions arise across the value chain, including enteric fermentation, manure management, feed production, land-use change, processing, transport, and packaging (Marandure et al. 2018). The principal GHGs are carbon dioxide (CO_2_), CH_4_, and N_2_O. CO_2_ is mainly associated with fossil fuel and energy use, whereas N_2_O results from soil nitrification and denitrification, and from manure decomposition, the latter of which also contributes to CH_4_ emissions (Pierrehumbert and Eshel 2015). Enteric CH_4_ is particularly important in GFB production systems because fibre-rich diets promote methanogenesis, and slower growth rates lengthen production cycles, increasing cumulative emissions (Doyle et al. 2023). Consequently, previous studies (Capper 2012; Klopatek et al. 2022a) report higher emissions per kg of beef from grass-fed systems than grain-fed systems. For example, Capper (2012) estimated 68% greater emissions in GFB. Mitigation strategies include improved grazing management, supplementation with crop residues or agro-industrial by-products, phytogenic diets that alter rumen microbial activity, and the use of dual-purpose or dairy cattle, in which emissions can be partly allocated to milk production (de Vries et al. 2015; Lagrange et al. 2021). Earlier slaughter may reduce lifetime emissions but raises quality and ethical concerns (Zhang et al. 2024).

Well-managed grasslands can sequester soil carbon and partly offset GFB-related emissions (Teague and Kreuter 2020; Wróbel et al. 2023), but the magnitude, permanence, and credibility of this offset remain contested (Molidor 2017). Regenerative grazing, silvopastoral systems, legume and multispecies pastures, biodiversity restoration, controlled burning, fertilisation, and improved grazing intensity can enhance carbon storage (Schuman et al. 2002; Henderson et al. 2015; Kleppel and Frank 2022). Grasslands can store about one-third of global carbon stocks, but sequestration varies with climate, soil, vegetation, geography, and management (Teague and Kreuter 2020; Wróbel et al. 2023). Dondini et al. (2023) estimated a global sequestration potential of approximately 300 kg C/ha/year over 20 years under soil organic carbon-enhancing management practices, with higher regional potential in Sub-Saharan Africa (410 kg C/ha/year). The influence of grazing intensity on carbon sequestration is also grassland-dependent, with positive effects in C4 grasslands and negative effects in C3 grasslands (Kleppel and Frank 2022).

Grazing can stimulate plant growth and carbon allocation to roots and soil microbes, enhancing soil carbon accumulation (Ren et al. 2024). It also influences methanotrophs, aerobic bacteria that oxidise CH_4_, by altering soil aeration, moisture, and compaction (Li et al. 2020). Light to moderate grazing may maintain or increase CH_4_ uptake, whereas heavy grazing and high stocking rates reduce methanotroph activity, increase emissions, and constrain soil carbon gains (Li et al. 2020; Purcell et al. 2025). However, N_2_O emissions from legume pastures, upstream input production, and downstream activities, including transport, processing and packaging, can offset sequestration benefits (FAO 2023). Notably, Putman et al. (2023) reported that over half of climate-related impacts occur upstream. Thus, carbon sequestration should complement, not replace, direct emission reductions (Henderson et al. 2015). Claims that GFB is climate-beneficial require caution because sequestration is temporary, context-specific, vulnerable to reversal by drought, overgrazing, or land-use change, and declines as soils approach carbon saturation. Credible climate benefits, therefore, depend on verifiable, long-term reductions in total and enteric emissions intensity.

#### Water usage

Water use is a key concern in beef production, particularly in water-scarce regions (Broom 2021). The water footprint (WF) includes green water (rainwater consumed or incorporated into products), blue water (surface and groundwater), and grey water (water needed to dilute pollutants; Gerbens-Leenes et al. 2013). Because green water is rainfall-derived, Broom (2019) argued that assessments should place greater emphasis on conserved blue and grey water. Low-input GFB production systems may therefore appear favourable, as they often depend on rainfall, whereas grain-fed systems typically have higher blue and grey WFs due to irrigated feed crops (Gerbens-Leenes et al. 2013; Broom 2019). However, GFB water use can increase substantially where pastures or fodder crops are irrigated. Broom (2019) ranked conserved water use highest in grain-fed systems, followed by fertilised irrigated pastures, natural pastures, and semi-intensive silvopastoral systems. Recent evidence from Argentina shows that extensive systems can have a higher total WF dominated by green water (98%), while intensive systems exhibit a lower total WF but greater grey-water shares (15–24%), linked to nitrogen leaching (Arrien et al. 2025). Hence, GFB water use is not inherently superior; outcomes depend on irrigation, pollution, nutrient losses, regional scarcity, and water opportunity costs.

#### Eutrophication and acidification

Evidence for the consistent eutrophication and acidification advantages of GFB over grain-fed beef remains limited, inconclusive, and context-dependent (de Vries et al. 2015). Nevertheless, concerns about synthetic fertilisers, pesticides, manure concentration, nutrient leaching, runoff, and soil acidification have led some to view the GFB production system as environmentally preferable to the grain-fed beef production system (Pierrehumbert and Eshel 2015; Molidor 2017). Grain-fed production systems depend on input-intensive crop production and tillage, which can contribute to soil carbon loss, erosion, biodiversity decline, and agrochemical runoff. By contrast, GFB production systems typically use fewer pesticides and fertilisers, potentially reducing pollutant loads (Cheung and McMahon 2017; Oerly et al. 2022). They also disperse excreta across pastures, improving nutrient cycling and reducing odour and waste-management risks associated with feedlots (McGinn et al. 2003; Hayek and Garrett 2018). Reduced feed production and transport may further lower smog formation potential (Klopatek et al. 2022a). The evidence suggests that GFB may reduce agrochemical impacts, but consistent evidence of eutrophication and acidification benefits remains insufficient to establish categorical superiority.

### Social quality

#### Animal welfare

Animal welfare relates to animals’ freedom from hunger, thirst, discomfort, pain, injury, disease, fear, and distress, and their ability to express natural behaviours (Davis et al. 2022). When forage is sufficient, GFB production systems are perceived to enhance welfare through pasture access, space, social contact, dietary choice, and expression of natural behaviour, aligning with consumer preferences for naturalness (Stampa et al. 2020; Broom 2021; Rivero and Lee 2022). Moreover, lower stocking densities may reduce stress and the risk of respiratory disease, while avoiding high-starch feedlot diets lowers the risk of acidosis and liver abscesses, potentially reducing antibiotic use (Sneeringer et al. 2015; Cameron and McAllister 2016; Carrillo et al. 2016). Polyphenol-rich legume pastures may reduce bloat by limiting foam formation, and may lower helminth burdens by impairing parasite development and reproduction, while supporting host nutrition and immune defence (Provenza et al. 2019; Lagrange et al. 2021; Macadam et al. 2022). However, extensive pastoral systems, especially in tropical regions, may compromise welfare through poor nutrition, water scarcity, parasitic diseases, exposure to adverse weather, predation, injuries, limited veterinary care, and infrequent monitoring (Marandure et al. 2020a; Abebe et al. 2022; Hernandez et al. 2022). Overall, welfare outcomes in GFB systems are context-dependent and influenced by the interaction between grazing-related benefits and extensive production constraints.

#### Meat safety

Meat safety refers to freedom from biological, chemical, and physical contaminants that may endanger human health and influence consumer purchasing decisions (Santos et al. 2021). Although GFB is often perceived as safer and more natural, the evidence supporting this view remains limited and context-specific (Lim et al. 2021). Earlier reviews found no consistent differences between grass- and grain-fed beef in total aerobic counts or generic *E. coli* (Mathews and Johnson 2013), while the higher microbial loads reported in some GFB samples appear to be linked more to processing-facility contamination than to the production system per se (Duarte et al. 2022). Antimicrobial resistance seems to provide a better distinction. Resistant *E. coli* may occur in both systems, but multidrug resistance and antibiotic-resistance genes are often higher in grain-fed cattle, particularly where ionophores are used (Zhang et al. 2010; Kwon et al. 2025). Restricted antibiotic and hormone use in GFB may also reduce residue-related concerns, supporting consumer willingness to pay premiums (Umberger et al. 2009; Sneeringer et al. 2015). Thus, meat safety advantages in GFB systems appear to be linked more strongly to antimicrobial stewardship and production practices than to feeding systems alone.

#### Traceability

Consumers are increasingly seeking products with traceable origins, driven mainly by food safety and provenance concerns (Lim et al. 2021). Support for local businesses remains an important credence attribute in purchasing decisions, reinforced by locavore movements that promote small-scale, low-input, locally produced GFB (Gwin et al. 2012; Apaoblaza et al. 2020). Traceability is therefore a major attribute in GFB certification and a feature for which consumers are often willing to pay more (Gwin 2009).

#### Tradition and culture

Grass-based cattle production is closely tied to the cultural identity of many pastoralist communities, where cattle serve not only as food and income but also as dowry, ritual assets, gifts, and inheritance (Mapiye et al. 2020; Oerly et al. 2022). These systems also help preserve locally adapted breeds such as Nguni cattle in Southern Africa, which are well suited to local environments but less attractive to feedlots because of lower growth rates and lighter carcass weights (Chingala et al. 2017; Mapiye et al. 2020). Hence, GFB systems can support both cultural heritage and livestock biodiversity, reinforcing their significance beyond agricultural production.

#### Cultural landscapes and agro-tourism

GFB farming helps preserve cultural landscapes and biodiversity while creating local economic opportunities, including agrotourism (Eriksson 2022). In sub-Saharan Africa and other parts of the world, scenic ranches, aesthetically distinctive breeds (e.g., Ankole and Nguni) and traditional production systems attract farm tourists (Marandure et al. 2020b; Oerly et al. 2022). Farm tours increase transparency, educate consumers, strengthen emotional ties to farms, diversify markets, and improve farmers’ livelihoods (Kline et al. 2016). This integration of agriculture, tourism, and cultural heritage underscores the broader socio-economic value of GFB systems in rural development.

#### Support for livelihoods and inclusive rural development

GFB can enhance social sustainability by supporting rural livelihoods, generating employment across the value chain, and strengthening the viability of livestock-dependent communities where alternative economic opportunities are limited (Abebe et al. 2022; Rivero and Lee 2022). By improving rural livelihoods, GFB production may also contribute to community stability, reduce rural-to-urban migration, and help preserve local cultures and traditions (Broom 2021). Beyond income generation, cattle provide liquid assets, manure, traction, insurance against shocks, and culturally important functions that strengthen social ties and resilience (Marandure et al. 2018; Mapiye et al. 2020). However, these benefits depend on broad and equitable participation across the value chain.

Participation in formal GFB value chains remains inequitable, particularly for smallholder and pastoralist producers, women, and the youth. Small-scale producers face low cattle offtake, high transaction costs, inadequate infrastructure, limited market information, and weak bargaining power, constraining their participation and value capture (Abebe et al. 2022; Mothupi 2025). In South Africa, In South Africa, communal producers’ limited engagement in formal livestock markets also highlights persistent weaknesses in market linkages and institutional support (Mothupi 2025). These structural barriers intersect with demographic and gender inequalities. Youth participation is constrained by unpaid herding responsibilities, limited access to land, capital, and training, and involvement primarily as labour rather than owners or decision-makers (Marandure et al. 2018). Women’s participation may enhance their social status, influence over resource governance, and access to support services, as demonstrated in GFB systems in the USA (Sitienei et al. 2020). However, women frequently remain disadvantaged in livestock ownership, decision-making, and market access; for example, their participation in communal cattle auctions in South Africa is limited even where female-headed households are prevalent (Mothupi 2025). Consequently, the social sustainability of GFB depends on equitable access to productive resources, markets, institutional support, and value-chain benefits. Inclusive policies, targeted capacity development, and strong producer organisations could reduce transaction costs, strengthen bargaining power, and support women’s empowerment and intergenerational succession among resource-constrained producers.

#### Quality of life and worker satisfaction and safety

Working in GFB value chains can be rewarding. Some studies have reported high self-rated quality of life among farmers producing GFB, while employees on silvopastoral beef farms in Colombia and Mexico reported greater job satisfaction and longer tenure than workers in grain-fed systems (Calle et al. 2009; McCutcheon et al. 2015). Moreover, worker safety may be better in small, slower-paced specialised GFB slaughterhouses than in high-throughput commercial abattoirs, where injury risk is higher (Gwin 2009).

#### Food security: feed-food competition and affordability

Food security depends not only on direct food production, but also on physical and economic access, income distribution, price stability, market functioning, and the availability of diverse foods (FAO et al. 2026). In this context, GFB production may indirectly contribute to food access by generating income and supporting livelihoods, rather than solely through direct meat consumption. GFB is often viewed as reducing feed-food competition because it largely uses marginal land and human-inedible feeds, although forage production may still occupy arable land in some regions (Tichenor et al. 2017a). Grain-fed systems face stronger criticism because they rely on human-edible grains that could directly support food security. While grain feeding increases productivity, grain-fed cattle may consume more human-edible protein than they produce (Thomas et al. 2021; Doyle et al. 2023; McGee et al. 2023). Australian evidence suggests both GFB and grain-fed production systems can generate positive net protein contributions, with GFB systems performing better (Thomas et al. 2021). Intensive systems can partly reduce competition by using agro-industrial byproducts and food waste, promoting circularity (Doyle et al. 2023). However, these feeds are also valuable in monogastric systems with higher conversion efficiency, creating cross-species competition. Thus, limiting the use of human-edible feeds and prioritising the efficient allocation of byproducts are essential for positioning GFB as a future-fit food system.

Affordability remains a key barrier to consumption, as higher production costs often require price premiums, at least 10% in some USA contexts, to maintain producer viability (Umberger et al. 2009; Rivero and Lee 2022). While such premiums benefit producers, they may limit access for low-income consumers and constrain market penetration, especially where consumers lack a clear understanding of GFB production systems and their associated product attributes, benefits, and trade-offs. Although information can improve attitudes and willingness to pay, knowledge gaps persist (Stampa et al. 2020; Santos et al. 2021). Targeted education and marketing could position GFB within a “less but better” consumption model, emphasising higher-value products consumed in smaller quantities.

#### Predation

Although cattle in GFB production systems enjoy freedom of movement, there is a high risk of predation, and especially of stock theft in Africa (Mapiye et al. 2009; Hadush 2021). This is one of the reasons why the number of grass feedlots is increasing in East Africa (Hadush 2021). Animal registration systems, community-based herding and guarding, stronger police-community collaboration, cross-border cooperation, and better animal identification and traceability are practical responses that may reduce these losses without resorting to permanent confinement (Mapiye et al. 2009).

### Economic quality

#### Profitability

GFB has emerged as a value-added niche in grain-fed beef markets, driven by consumer demand for safer, healthier, and ethically produced meat (Marandure et al. 2018; Stampa et al. 2020). Evidence indicates that, where grain prices are high and/or pastures are well managed, GFB production systems reduce feed costs and improve gross margins relative to grain-fed systems (Cheung and McMahon 2017; Greenwood 2021; Castaño-Sánchez et al. 2023; Doyle et al. 2023). However, GFB profitability depends primarily on securing price premiums supported by differentiation, traceability, credible labelling, niche marketing, and direct market access (Gwin 2009; Cheung and McMahon 2017; Torell et al. 2023). Pasture quality, input costs, breed choice, and finishing strategy further influence profitability. Drought-adapted breeds such as Nguni (Mapiye et al. 2020) and Raramuri (Castaño-Sánchez et al. 2023) may reduce feed costs, which account for more than 60% of production costs, while cultivated pastures can outperform natural pasture and grain finishing (Pashaei Kamali et al. 2016). However, the benefits of GFB production systems may be offset by longer finishing periods, greater land requirements, climate variability, certification and traceability costs, labour demands, disease pressure, and the loss of premiums when standards are not maintained (Qushim et al. 2018; Klopatek et al. 2022a).

#### Supply chain constraints

Distribution remains a key constraint in the GFB sector because transport, processing, storage, and marketing infrastructure, especially in developed countries, were designed around cheaper grain-fed beef, limiting access to dedicated supply chains and specialised processors (Cheung and McMahon 2017; Poore et al. 2020). Moreover, carcass valuation systems, including in Southern Africa, often favour younger grain-fed cattle and undervalue grass-fed carcasses (Chingala et al. 2017). These barriers can be partly addressed through product differentiation, certification, direct marketing, and traceability, which strengthen market identity and support consumer willingness to pay GFB premiums (Gwin 2009).

#### Product supply and quality consistency

Ensuring a consistent year-round supply of GFB with uniform quality is challenging because seasonal and climate-driven fluctuations in pasture biomass and nutritive value affect growth, breeding, finishing duration, carcass traits, and meat quality, creating greater variability than grain-finishing systems (Krusinski et al. 2022b). Accordingly, supply consistency is likely to be more attainable in climatically favourable regions compared to drought-prone areas (Castaño-Sánchez et al. 2023). Mineral licks, conserved forages, and targeted supplementation in drought-prone areas can reduce feed gaps, but may alter beef nutritional and bioactive profiles and, in some cases, conflict with certification requirements (Krusinski et al. 2022a, b; Doyle et al. 2023). In tropical developing regions, including in Sub-Saharan Africa, smallholders face additional constraints related to drought, management capacity, land tenure, extension, and markets (Marandure et al. 2018; Abebe et al. 2022). Supporting producer cooperatives to aggregate livestock and other resources could increase marketable volumes and improve supply consistency. This should be complemented by consumer education on GFB seasonality to facilitate supply coordination and manage expectations regarding product availability.

#### Scalability of grass-fed beef operations

As demand grows, scaling up GFB operations is increasingly important but constrained by limited access to finance, affordable land, suitable breeds, feed, and water (Gwin 2009; Cheung and McMahon 2017). Expansion further presents the challenge of increasing production speed, volume, and consistency while preserving core values, namely animal welfare, environmental quality, and human health, and maintaining affordability (Gwin 2009). Sustainable scaling, therefore, depends on strong third-party standards, smallholder producer networks, investment in local processing, supportive land-use policies, subsidy reform, and regional collaboration (Gwin 2009; Cheung and McMahon 2017).

## Assessments of grass-fed beef sustainability

Although interest in GFB has generated empirical evidence regarding its sustainability attributes, appropriate assessment frameworks are still lacking. Achieving sustainability requires an integrated balance of environmental, social, and economic pillars throughout the value chain, while accounting for interdependencies, trade-offs, and feedback loops (Oerly et al. 2022; Rivero and Lee 2022). Table 4 synthesises 21 studies, detailing the pillars evaluated and methods applied.

### Assessment methods and indicators

Across the 21 studies comparing grass-fed and grain-fed beef production systems (Table 4), sustainability assessment was uneven across pillars. Environmental indicators were predominant, featured exclusively in 10 studies and absent from only one. Six studies combined environmental and economic indicators, while economic assessment alone appeared in one study and environmental-social assessment in another. Only three studies integrated environmental, economic, and social dimensions, indicating the limited use of holistic sustainability frameworks. This imbalance supports de Vries et al. (2015), who observed that comparative beef-system studies often omit key sustainability impact categories, limiting comprehensive evaluation.

**Table 4:** Studies involving sustainability assessments of grass-fed beef production systems

Pillar assessed	Number of studies	Type of assessment methods used	Reference
Environment only	10	Life Cycle Assessment; Integrated Farm System Model; BEEF Greenhouse Gas Emissions Model; Land-use ratio calculations	Wiedemann et al. (2015), Australia Tsutsumi et al. (2016), Japan Tichenor et al. (2017a), USA Tichenor et al. (2017b), USA Rowntree et al. (2016), USA Rowntree et al. (2020), USA Putman et al. (2023), USA Pelletier et al. (2010), USA Macadam et al. (2022), USA Modernel et al. (2013), Uruguay
Economic only	1	Profitability analysis	Torell et al. (2023), USA
Economic and environment	6	Profitability analysis; Life Cycle Assessment; Grange Scottish Beef Model; Agricultural Resource Efficiency Calculator	Bhandari et al. (2015), USA Castaño-Sánchez et al. (2023), USA Kamilaris et al. (2020), Scotland Klopatek et al. (2022a), USA Pashaei Kamali et al. (2016), Brazil Stanley et al. (2018), USA
Environment and social	1	GrassGro^®^ biophysical model	Thomas et al. (2021), Australia
Economic, social, and environment	3	System dynamics; BEEF systems Greenhouse gas Emissions Model (BEEFGEM); Profitability analysis; Chemical analysis	Marandure et al. (2020b), South Africa McGee et al. (2023), Ireland Doyle et al. (2023), Ireland

Life cycle assessment (LCA) remains the dominant method for environmental assessment in beef studies, often complemented by farm-level models such as the Grange Scottish Beef Model, the Integrated Farm System Model, the BEEFGEM, and GrassGro^®^. Most studies in Table 4 assessed impacts only up to farmgate or finishing, with Wiedemann et al. (2015) extending to retail and Putman et al. (2023) covering the full life cycle. Although cradle-to-farmgate assessments are common, broader value-chain analyses offer more complete sustainability insights and reduce the risk of burden-shifting (Putman et al. 2023).

Functional-unit selection substantially shapes the comparative sustainability outcomes of GFB assessments because it determines how impacts are expressed (Fan et al. 2022). Kilogram-of-beef metrics typically emphasise production efficiency, whereas land-, human-edible protein-, nutritional-value-, or economic-based units may favour systems delivering broader ecological, nutritional, or socioeconomic benefits. Consequently, divergent findings across studies may reflect different sustainability objectives rather than methodological inconsistency. Functional units should therefore align with the assessment objective, be reported transparently, and, where feasible, be supplemented with alternative units to reveal trade-offs and avoid conclusions driven by a single denominator.

Within the environmental dimension, seven studies (Bhandari et al. 2015; Pashaei Kamali et al. 2016; Tsutsumi et al. 2016; Tichenor et al. 2017b; Rowntree et al. 2020; Klopatek et al. 2022a; Putman et al. 2023) assessed carbon-related emissions using global warming potential (GWP), which converts the heat-trapping effects of GHGs into CO_2_-equivalents (CO_2_-eq) over a defined time horizon relative to CO_2_. The 100-year metric (GWP100), widely adopted in climate policy and LCA, was used in the three most recent studies (Rowntree et al. 2020; Klopatek et al. 2022a; Putman et al. 2023). This metric may misrepresent CH_4_-intensive pasture-based systems because it does not fully reflect differences in atmospheric lifetimes, given that CH₄ is short-lived (~ 12 years). In comparison, N₂O persists for ~ 110 years and CO_2_ for centuries to millennia (McAuliffe et al. 2023). Although CH_4_ has a GWP100 of ~ 28 CO_2_-eq, its warming effect depends strongly on whether emissions are increasing, stable, or declining. Alternative metrics, such as GWP^*^, express emissions as CO₂-warming equivalents by linking short-lived climate pollutants to changes in emission rates, thereby better capturing their temperature effects (McAuliffe et al. 2023). None of the studies in Table 4 applied GWP^*^, highlighting a key limitation and research gap for GFB systems.

Traditional LCAs often exclude soil carbon sequestration, producing incomplete beef carbon footprints, especially in arid systems where sequestration remains uncertain and difficult to quantify (Castaño-Sánchez et al. 2023). When sequestration is included, sustainability assessments report markedly lower footprints for GFB (Picasso et al. 2014; Rowntree et al. 2016; Stanley et al. 2018). Notably, Stanley et al. (2018) found that GFB had 158% higher GWP without sequestration but became net negative when sequestration was considered.

The social pillar remains underexplored, with only four studies including social indicators (Table 4). Animal welfare and nutritional/health attributes, key drivers of GFB sectoral growth, were rarely assessed. Notably, nutrition was considered in only two studies (Doyle et al. 2023; McGee et al. 2023), through nutritional indices and meat quality. Beyond food security, GFB may enhance rural livelihoods, employment, smallholder participation, community identity, consumer trust, and welfare-oriented value chains. However, equity, labour quality, gender, affordability, traceability, and local impacts are often overlooked, reflecting weak social assessment frameworks (Gaviglio et al. 2016). Future assessments should use participatory, context-specific social indicators and support fair, inclusive markets.

### Interrelationships and trade-offs between sustainability components

Trade-offs between profitability and GHG emissions are evident across GFB production systems (Bhandari et al. 2015). Kamilaris et al. (2020) found that while grain-fed beef production systems yielded lower negative environmental impacts, medium-duration (18–24 months) GFB production systems in Scotland effectively balanced environmental performance with financial benefits. Torell et al. (2023) highlighted that their unpublished data show that the crossbreed cattle that performed better economically required less water but had a longer lifespan, which might imply greater net GHG emissions. Within the environmental pillar, Tichenor et al. (2017a) demonstrated that although lower carbon and land footprints are often reported in grain-fed beef production systems, they often have higher eutrophication and acidification impacts per unit of land compared to GFB production systems. While feedlot finishing lowers enteric GHG emissions, the manufacturing of grain-based feeds is emission-intensive. As a result, there are potential trade-offs between enteric CH_4_ and N_2_O emissions from feed manufacturing (Kamilaris et al. 2020).

McGee et al. (2023) found that, while GFB production systems are more efficient in terms of the amount of human-edible food used to produce meat, grain-fed beef production systems are more productive in terms of meat yield per hectare. Trade-offs also exist between animal welfare and environmental efficiency. For example, welfare-oriented GFB production systems often require more land, while grain-fed production systems, although land-intensive, may restrict natural behaviours and increase the risk of bovine respiratory illnesses (Hadush 2021; Broom 2021). In addition, there are trade-offs between animal welfare (freedom of movement) and the risks of predation and stock theft in GFB production systems. Overall, such animal welfare-related trade-offs may be more pronounced in extensive rangeland systems in developing regions than in temperate pasture systems in developed regions. It is imperative to note that the interrelationships among the sustainability pillars are not only dynamic but also evolve over time as changes in system conditions, resource availability, and management practices alter the strength and direction of interactions among the pillars. These interactions are mediated by feedback loops, through which changes in one sustainability dimension can influence others and, in turn, shape the subsequent evolution of GFB production systems (Marandure et al. 2020b). Except for systems dynamics (Marandure et al. 2020b), most of the tools used do not capture these aspects, interrelationships, and trade-offs.

### Implications of grass-fed beef sustainability assessments for tropical production systems

GFB sustainability assessments in the tropics should move beyond temperate, high-input assumptions that ineffectively reflect local breeds, forage dynamics, production objectives, and market realities. Locally adapted cattle, including *B. indicus*, *B. taurus africanus* and indigenous composite breeds, should not be penalised through generic productivity benchmarks. Instead, assessments should recognise adaptation traits such as drought resilience, heat tolerance, disease and parasite resilience, and survival efficiency. Under tropical conditions, static LCA may be insufficient because seasonal forage availability differentially affects rangeland condition, CH_4_ intensity, animal growth, health, and welfare. Functional-unit choice is also critical, as tropical rangelands provide biodiversity, carbon storage, cultural value, and livelihood resilience beyond beef production. Relying solely on product-based units may undervalue the multifunctional benefits of rangelands and locally-adapted cattle, producing incomplete or misleading sustainability comparisons. Thus, tropical GFB systems evaluations should be dynamic, integrating seasonal feed deficits, forage banks, adaptive stocking rates, and drought contingency planning to better reflect real production risks.

Smallholder systems, which are dominant in the tropics, are often misclassified as inefficient when informal labour, manure recycling, household nutrition, socio-cultural value, risk buffering, and multifunctionality are excluded from the assessment. Participatory, low-cost, and easy-to-measure indicators are therefore critical for generating inclusive and context-sensitive sustainability assessments that better reflect the realities, priorities, and constraints of tropical livestock production systems. Under harsher future climates, GFB production has the potential to remain viable in tropical systems through the adoption of adaptive practices that enhance resilience. Silvopastoral systems and diverse pastures may provide shade, biodiversity, carbon storage, and greater resilience to heat and rainfall variability. The environmental benefits should be weighed against animal-welfare risks. Continuous access to pasture may expose cattle to severe heat stress, water scarcity, and parasitic infections, particularly during hot and dry seasons (Abebe et al. 2022; Hernandez et al. 2022). Accordingly, assessments should consider these animal-welfare risks. Moreover, soil-carbon sequestration should not be regarded as a permanent offset to CH₄ emissions, because warming can stimulate the decomposition and loss of previously stored soil carbon, while seasonal drying followed by rewetting can produce short-lived pulses of carbon mineralisation and CO₂ release (McFarlane et al. 2024; Ren et al. 2024). Carbon benefits should therefore be supported by long-term, site-specific measurements of carbon stocks and fluxes.

In tropical livestock systems, limited market access can prevent sustainability premiums from reaching producers in informal or weakly integrated value chains. Sustainability assessments should therefore account for producer cooperative organisation, local procurement, inclusive value chain partnerships, market inclusion, and benefit-sharing mechanisms to ensure that sustainability gains translate into tangible livelihood benefits. Traceability and certification in the tropics face practical challenges that often marginalise sustainable producers, including poor infrastructure, communal grazing, fragmented herds, expensive audits, and limited records. These challenges highlight the need for affordable digital records, group certification and context-sensitive verification and integration in sustainability assessments.

## Study limitations, research gaps, actionable insights for the industry and future directions

Evidence of GFB sustainability remains difficult to synthesise for decision-making. The literature is geographically imbalanced, with Africa and Asia underrepresented despite extensive pasture-based finishing systems, likely reflecting indexing bias, fragmented publication outlets, and inconsistent terminology rather than limited production. This gap is important because sustainability outcomes are dependent on geography, climate, forage availability, management practices, breed composition, institutional arrangements, market access, and stakeholder priorities. Consequently, conclusions drawn from temperate production systems cannot be assumed to apply directly to tropical or subtropical regions. Evidence is also concentrated at the farm level, while post-farmgate stages are rarely assessed, although they influence whole-chain environmental burdens, product quality, costs, and consumer value. Moreover, the absence of a standardised definition of GFB, compounded by heterogeneity in forage species diversity, nutritional and bioactive composition, digestibility and seasonal availability, supplementation practices, animal genetics, management systems, and production environments, limits cross-study comparability and contributes to variation in reported sustainability outcomes.

Major research gaps include the lack of context-specific, whole-value-chain assessments that integrate environmental, economic, and social dimensions. Sustainability comparisons between GFB and grain-fed beef are meaningful only when functional units, system boundaries, production periods, allocation procedures, and impact-assessment methods are clearly defined and consistently applied. Current studies often use narrow indicator sets, inconsistent system boundaries, and different functional units (Wiedemann et al. 2015; Doyle et al. 2023), obscuring trade-offs among GHG emissions, soil health, biodiversity, pesticide risk, animal welfare, carcass value, nutrition, and eating quality (Modernel et al. 2013; Broom 2021; Moloney et al. 2022). Existing frameworks also insufficiently capture seasonal pasture variability, product supply consistency, methane dynamics, GWP^*^ effects in CH_4_-intensive systems, feedback loops, ecosystem services, and demand drivers such as health perceptions, safety, welfare, and socio-cultural values (Marandure et al. 2018; McAuliffe et al. 2023). Some climate impacts may relate more to ecosystem services than to meat output, suggesting scope to value animal products partly for their environmental benefits (Lobato et al. 2014).

For industry, improving GFB competitiveness requires producer collaboration to build scale, lower unit costs, strengthen input purchasing, access specialised services, improve bargaining power, and enter higher-value markets. Strategic forage planning should align calving and finishing with seasonal pasture peaks and be supported by conserved forages during feed gaps (Poore et al. 2020). Adaptive grazing, locally-adapted multispecies swards, and plant by-product supplements that stabilise forage supply and quality require scaling (Krusinski et al. 2022b). Carcass valuation systems designed for grain-fed beef should be revised or complemented to reward GFB desirable quality attributes, drawing on schemes such as Meat Standards Australia (Chingala et al. 2017). Literature reviews should avoid unsupported human-health claims and focus on verified compositional benefits unless clinical trials confirm disease-risk effects. Holistic sustainability assessments of GFB require transparent, stakeholder-informed frameworks that integrate multidimensional indicators, trade-offs, and value chain dynamics using complementary tools such as LCA and system dynamics.

## Conclusions

Current conflicting evidence does not justify that GFB is inherently sustainable. Well-managed grazing systems and diverse pastures may improve animal welfare, biodiversity, beef FA composition, flavour, and bioactive profiles. However, these advantages can be offset by greater land requirements, slower growth rates, darker meat colour, variable tenderness, inconsistent CH_4_ intensity, uncertain carbon sequestration, and irregular product supply. Sustainability outcomes are also context-specific, varying with management practices, breeds, climate, and regional market conditions. They are also materially influenced by methodological choices, including the selection of metrics, system boundaries, and functional units. Therefore, policymakers and standard-setting bodies should not evaluate GFB solely against conventional grain-fed beef benchmarks. Rather, sustainability assessment frameworks should integrate environmental and economic indicators with social metrics such as nutrition, human health value, and animal welfare using a value chain approach and clearly defined functional units and system boundaries. In practice, collaborative producer networks, including cooperatives, should be established to strengthen distribution, supply consistency, traceability, processing capacity, certification, and market access. Future research should prioritise the development of context-appropriate, value chain-based multidimensional sustainability evaluation tools to support credible authenticity claims, targeted incentives, and sustainable market development for GFB systems.

## Supplementary Information

Below is the link to the electronic supplementary material.

Supplementary Material 1

## Data Availability

No data was used for the research described in the article.
