# Supplementary material for: Rethinking the sustainability assessment of grass-fed beef: a holistic systematic review
Source: Trop Anim Health Prod. 2026 Nov 4;58(8):641. doi: 10.1007/s11250-026-05471-2 (PMC13736477; doi:10.1007/s11250-026-05471-2)
Supplement: Supplementary file 1 [file 11250_2026_5471_MOESM1_ESM.docx]

**Table S1 Search strings applied in the electronic databases**

| **Database** | **Search string^*^** |
| --- | --- |
| Scopus | ALL ( ( ( "grass-fed" OR "grass-finished" OR "pasture-fed" OR "pasture-finished" OR "forage-fed" OR "forage-finished" ) AND ( beef OR steer OR sickle OR bull OR cattle OR heifer OR cow ) ) AND ( production OR supply AND "management practices" AND perceptions ) OR ( consumption OR demand OR "willingness to pay" AND perceptions ) OR ( market* OR "marketing practices" ) OR ( quality OR "credence attributes" ) OR ( "environmental contribution" OR "environmental impact" OR "environmental consequences" OR "environmental assess*" OR "social impact" OR "social contribution" OR "economic contribution" OR "economic impact" OR "economic performance" OR sustainability ) OR ( challenges OR opportunities ) ) AND PUBYEAR > 1999 AND PUBYEAR < 2027 AND ( LIMIT-TO ( DOCTYPE , "ar" ) ) AND ( LIMIT-TO ( LANGUAGE , "English" ) ) |
| Web of Science | TS=((("grass-fed" OR "grass-finished" OR "pasture-fed" OR "pasture-finished" OR "forage-fed" OR "forage-finished") AND (beef OR steer OR sucker OR bull OR cattle OR heifer OR cow)) AND ((production OR supply OR "management practices" OR perceptions) OR (consumption OR demand OR "willingness to pay" OR perceptions) OR (market* OR "marketing practices") OR (quality OR "credence attributes") OR ("environmental contribution" OR "environmental impact" OR "environmental consequences" OR "environmental assess*" OR "social impact" OR "social contribution" OR "economic contribution" OR "economic impact" OR "economic performance" OR sustainability ) OR (challenges OR opportunities))) |
| Agricola | Any field contains (("grass-fed" OR "grass-finished" OR "pasture-fed" OR "pasture-finished" OR "forage-fed" OR "forage-finished") AND (beef OR steer OR sucker OR bull OR cattle OR heifer OR cow)) AND ((production OR supply OR "management practices" OR perceptions) OR (consumption OR demand OR "willingness to pay" OR perceptions) OR (market* OR "marketing practices") OR (quality OR "credence attributes") OR ("environmental contribution" OR "environmental impact" OR "environmental consequences" OR "environmental assess*" OR "social impact" OR "social contribution" OR "economic contribution" OR "economic impact" OR "economic performance" OR sustainability ) OR (challenges OR opportunities)) |

^*^ For Web of Science and Agricola, results were filtered to English-language records published from 2000 onwards.
